# Polymers Enhancing Bioavailability in Drug Delivery, 2nd Edition

**DOI:** 10.3390/pharmaceutics15112604

**Published:** 2023-11-08

**Authors:** Ana I. Fernandes

**Affiliations:** Egas Moniz Center for Interdisciplinary Research (CiiEM), Egas Moniz School of Health & Science, 2829-511 Caparica, Portugal; aifernandes@egasmoniz.edu.pt; Tel.: +351-212946823

This Special Issue continues the previously published work [1] with the aim of updating the current status and presenting the emerging trends in polymeric drug delivery systems which are designed to improve drug bioavailability. As discussed there, many drugs are poorly bioavailable due to their low water solubility and/or low permeability across epithelia. Others are labile when administered orally due to acidic pHs or enzymatic degradation, precluding their use by the route most preferred by patients. Still, others, although administered through routes with reduced proteolytic activity, lack specificity and may require localization strategies at the site of action to maximize their therapeutic index. The use of polymers in drug delivery is well established [2,3,4] and essential to achieve improved drug pharmacokinetics and pharmacodynamics, as well as patient compliance [5].

A total of 12 original research papers and 1 literature review were published with open access; 21 papers were submitted for publication, representing an acceptance rate of 61.9%, ensuring the publication of high-quality work.

The current issue reflects the diversity of approaches from different research groups, ranging from drug encapsulation in nanostructures (e.g., nanoparticles, nanocrystals, nanosponges, micelles, dendrimers) to drug amorphization, co-amorphization or 3D-printed scaffolds for local drug delivery. Polymers are also used as hydrophilic coatings to provide biocompatibility or antimicrobial properties and are grafted/functionalized for target-specific delivery, as well as to provide stimuli-responsive controlled release of the drug. In fact, many of the reported strategies involve the synthesis of novel polymers/copolymers or the chemical conjugation of residues to tailor drug release or provide recognition (active targeting) or biomimetic or stealth (long blood circulation, passive targeting) properties, highlighting the importance of the chemical engineering of polymers [6] to improve performance.

The routes of administration of the systems described in this Special Issue are summarized in Figure 1. Oral and parenteral routes are the most common (nine publications); noteworthy is the representation (two papers) of medical devices such as catheters and scaffolds. Antimicrobials, antifungals, cytotoxics, anti-inflammatories, antidiabetics and immunosuppressants are among the investigated drugs.

An overview of the strategies described to treat and diagnose diseases or to provide advanced theranostic applications is given below.

The use of colloidal systems is explored in the following set of works. Itraconazole was encapsulated in core–shell-like polymeric nanoparticles functionalized with antibodies to provide both controlled release of the antifungal and targeting of macrophages. Drug-loaded poly-(lactic-co-glycolic acid) (PLGA, one of the most successful biodegradable polymers [7]) nanoparticles were prepared using a high-energy emulsification method and then covalently coupled to the antibodies using carbodiimide; their size was estimated as 226.66 ± 13.05 nm and their surface ζ-potential was estimated as −27.9 ± 0.26 mV, features that ensure a lack of particle aggregation and clearance by the mononuclear phagocytic system. The strategy provided improved drug stability, solubility and bioavailability, thus requiring lower doses for antifungal activity. Histoplasmosis was used as a model of intracellular infection, and antibody-directed nanoparticles showed significantly higher uptake in vitro by murine cells compared to bare nanoparticles, with no cytotoxic effect on macrophages. They also induced the elimination of *Histoplasma capsulatum*, an intracellular fungus, in co-cultures. In addition, the gene expression of anti-inflammatory and pro-inflammatory cytokines (IL-1, INF-γ, IL-6 and IL-10) on macrophages was modulated by the encapsulated drug, and the antibody coating provided an enhanced natural cellular response, synergistically preventing fungal growth at the intracellular level. Intracellular drug delivery is very promising since it reduces resistance to pathogens and prevents non-specific accumulation in other tissues, thereby reducing toxicity and side effects.

Superparamagnetic iron oxide nanoparticles (SPIONs), approved for diagnosis and treatment of malignant tumors, are biodegradable but require coating with biocompatible, non-cytotoxic materials to prevent embolism. In this work, a biocompatible copolyester—poly(globalide-co-ε-caprolactone) (PGICL)—was synthesized and then modified with cysteine (Cys) via a thiol-ene reaction to obtain PGlCLCys. Due to the reduced crystallinity and increased hydrophilicity, the Cys-modified copolymer was used to coat the SPIONs. The free amine groups of Cys on the particles’ surface were conjugated either to folic acid (targeting moiety to promote internalization, since receptors are overexpressed in many tumors) or methotrexate (targeting residue because it also specifically interacts with folate receptors and anti-cancer drug by blocking the use of folic acid by the body) with conjugation efficiencies of 62% and 60%, respectively. Protease incubation under mimicked lysosomal conditions (37 °C in phosphate buffer pH ~5.3, 72 h) resulted in the enzymatic release of 45% of the particle-conjugated drug. The breast carcinoma cell viability was reduced by 25% (MTT assay) after 72h of contact. The successful conjugation and triggered release of methotrexate in this model multifunctional platform pave the way for the development of more selective and less aggressive cancer treatments and/or diagnostics.

Neuroprotective particles targeted to the retina, developed by Colucci et al., have shown promise for the treatment of retinal degeneration. The retina is a highly metabolic tissue, very much affected by oxidative stress. The nerve growth factor (NGF) is a neurotrophin involved in the molecular response to oxidative damage. The neuroprotective and regenerative effects of the NGF are hampered by rapid degradation and clearance in vivo. Moreover, the anatomical and physiological barriers that protect the eye make the delivery of drugs to the posterior segment challenging, even using nanodevices which have been shown to increase the bioavailability of drugs. In this work, polyacrylamide nanoparticles (ANPs) were noncovalently functionalized with peanut agglutinin (PNA) for targeting and NGF for neuroprotection. The ANP:PNA:NGF nanoparticles were synthesized and characterized, and their bioactivity and protective effects were evaluated in vitro. The teleost zebrafish (*Danio rerio*) was used as an in vivo model organism to evaluate this new ocular therapeutic strategy. The nanoformulation (22.81 ± 3.17 nm) was targeted and demonstrated a prolonged residence time in the posterior segment of the eye; it was also shown to preserve human retinal cell viability under oxidative conditions and improve the visual function of zebrafish larvae, partially reducing oxidative-stress-triggered retinal cell apoptosis. The intravitreal administration of this nanosystem could overcome the need for multiple injections due to the unfavorable kinetics of the usual form of the drug, thereby reducing side effects.

Colon delivery of budesonide in nanoparticles was attempted to circumvent the limitations of oral administration [8]. Targeting topical anti-inflammatory drugs to the colon is effective in the treatment of inflammatory bowel diseases. Inflamed tissues accumulate nanoparticles due to the loosening of tight junctions. Chitosan (CS) is a biodegradable cationic polysaccharide with mucoadhesive properties to the negatively charged intestinal mucosa. As such, in this work, CS nanoparticles were loaded with budesonide by an ionic gelation technique, and characterized in terms of size, shape, ζ-potential, encapsulation efficiency and drug release. Nanoparticles were then pelletized by extrusion–spheronization and the pellets were coated with (1) two enteric polymers (Eudragit^®^ L and S) to minimize early drug release in the upper GI tract and (2) time-dependent polymers (Eudragit^®^ RS) for colon targeting. The size, morphology and mechanical properties of the pellets and in vitro drug release characteristics were evaluated. Wistar rats with induced colitis were used to evaluate the anti-inflammatory effect of the formulation and the relapse time after treatment discontinuation. Pelletized budesonide nanoparticles exhibited faster drug release in acidic pH; drug release in the GI tract was sustained but incomplete; and the anti-inflammatory effect lasted longer compared to conventional budesonide pellets.

Rutin is a plant flavonoid with a wide spectrum of clinical applications limited by a low solubility and bioavailability. To overcome these limitations, rutin nanocrystals (submicron colloidal dispersions of 100% drug) were prepared by the anti-solvent nanoprecipitation–ultrasonication method using a variety of stabilizers, including non-ionic surfactants and non-ionic polymers. The nanocrystals were characterized regarding their particle size, size distribution, morphology, ζ-potential, entrapment efficiency, colloidal stability, rutin photostability, dissolution rate and saturation solubility. After dispersion of the crystals in a hydroxypropyl methyl cellulose hydrogel base, the drug release kinetics and permeability through mouse skin were evaluated. The anti-inflammatory efficacy of rutin was investigated in a carrageenan-induced rat paw edema model. The size of the nanocrystals ranged from 270 to 500 nm with a polydispersity index of about 0.3–0.5. The size of the nanocrystals increased with storage time and the photostability of the rutin was improved approximately 2.3-fold. The use of hydroxypropyl beta-cyclodextrin (HP-β-CD) as a stabilizer resulted in the smallest nanocrystals size, the best colloidal stability, the highest drug entrapment efficiency and the highest drug photostability. Depending on the stabilizer, the aqueous solubility and dissolution rate of the drug were increased by 102- to 202-fold and 2.3- to 6.7-fold, respectively. Compared to the free drug hydrogel, a significantly higher percentage of drug was released from the HP-β-CD nanocrystal hydrogel and permeated through the mouse skin; the cumulative amount of drug penetration of the skin was 2.5 times higher. In vivo edema inhibition was also significantly higher than with the free rutin hydrogel and commercial diclofenac sodium gel. This study demonstrated the potential of nanocrystals to improve the solubility, dissolution rate and anti-inflammatory properties of drugs, and highlighted the importance of careful stabilizer selection.

Targeted β-cyclodextrin nanosponges containing fisetin have been developed for the effective management of breast cancer. Fisetin is a phytomedicine with a therapeutic potential that is significantly reduced by a low systemic bioavailability due to its highly lipophilic nature. In this work, lactoferrin-coated FS-loaded β-cyclodextrin nanosponges (LF-FS-NSs) were developed in an attempt to circumvent such a limitation. Nanosponges were produced through cross-linking of β-cyclodextrin by diphenyl carbonate and coated with lactoferrin for active targeting to transferrin receptors overexpressed in breast cancer cells. Fourier transform infrared (FTIR) and X-ray diffraction (XRD) confirmed the presence of nanosponges. Scanning electron microscopy (SEM) analysis revealed its mesoporous spherical structure with pore diameters of ~30nm, which was further confirmed by surface area determination. The selected LF-FS-NSs presented high loading efficiencies (96 ± 0.3%), good colloidal properties (size 52.7 ± 7.2 nm, dispersion index <0.3, and ζ-potential 24 mV) and Fickian diffusion-controlled sustained drug release. The oral and intraperitoneal bioavailability of nanosponge-encapsulated fisetin was enhanced in rats (2.5- and 3.2-fold, respectively) compared to drug suspension rats. The antitumor efficacy of LF-FS-NSs evaluated in vitro (MDA-MB-231 cells) and in vivo (Ehrlich ascites mouse model) was significantly higher due to their superior activity and targetability compared to the free drug and uncoated formulation. The authors concluded that the formulation is promising for the effective management of breast cancer.

Novel hybrid block copolymers have been synthesized through ring-opening polymerization and used in the production of self-assembling micelles incorporating doxorubicin. The hybrid amphiphilic copolypeptides were synthesized from poly(ethylene oxide) (PEO), poly(l-histidine) (PHis) and poly(l-cysteine) (PCys). In aqueous media, they form micelles with an outer hydrophilic PEO corona and a redox- and pH-responsive hydrophobic layer, respectively, due to PHis and PCys. Crosslinking of PCys’s thiol groups further stabilized the nanoparticles. Structural characterization using dynamic and static light scattering and transmission electron microscopy (TEM) revealed mainly a core–shell micellar structure. Structure and conformation studies of synthetic polypeptides were performed by circular dichroism; the neutral ζ-potential of particles indicated that PEO blocks were the outer, stealthy layer. Doxorubicin was encapsulated in the hydrophobic core and released in response to changes in pH and redox (using glutathione, which is elevated in tumors) mimicking the tumor microenvironment. The topology of PCys (either the middle block, the end block or randomly distributed along the PHis chain) significantly affected the structure of the micelles and the drug release profile. The antiproliferative activity of the doxorubicin-loaded nanocarriers, tested in three different breast cancer cell lines, was comparable to that of the free drug.

Insulin delivery to treat diabetes mellitus was attempted through pegylated ferrisilicate with large 3D pores. Due to the large surface area (804 m^2^/g) and cubic pores (3.2 nm), the material was explored to improve drug encapsulation and loading efficiency. Ferrisilicate was coated with 400 D polyethylene glycol (PEG) to enhance drug stability and permeability across intestinal epithelia and provide pH sensitivity. The characterization methods included XRD, BET surface area analysis, FTIR and TEM analysis. After optimizing the insulin load, its release mechanism was studied using the dialysis membrane (MWCO = 14,000 Da) technique at pHs of 1.2, 6.8 and 7.4. The Korsmeyer–Peppas model was used to evaluate the kinetics and mechanism of drug release at different pH values. The in vitro cytotoxicity of the nanoformulations, evaluated in human foreskin fibroblast (HFF-1) cells using a 3-(4,5-dimethylthiazol-2-yl)-2,5-diphenyltetrazolium bromide (MTT) assay, was very low. On acute oral administration to diabetic Wistar rats, insulin/ferrisilicate/PEG (doses of 5 and 10 mg/kg body weight) demonstrated a hypoglycemic effect, significantly reducing blood glucose levels compared to the control. The insulin encapsulation/loading capacity (46%) and the smart, pH-sensitive kinetic release behavior observed demonstrate the potential of the system.

The use of nanosized Janus and dendrimer particles for the targeted delivery and enhanced bioavailability of pharmaceuticals has been extensively reviewed. The high surface-to-volume ratio and surface-charge-dependent behavior, as well as size- and shape-dependent tailorable properties, make nanoparticles particularly attractive for drug delivery. Janus nanoparticles are composed of two distinct components that differ in physical and chemical properties, representing a unique platform for the co-delivery of multiple drugs or the targeting of specific tissues for treatment and/or diagnosis of diseases. Their complex synthesis and residual solvent toxicity are significant limitations to their use. On the other hand, dendrimers are monodisperse, hyperbranched synthetic macromolecules with well-defined functional groups on the surface, and can be engineered for improved drug targeting (e.g., overexpressed receptors in tumors) and release. Scalability issues, a high non-specific toxicity and a low hydro-solubility are dendrimer-associated drawbacks. Despite the limitations, both nanocarriers are capable of improving drug solubility in water and drug stability, increasing the intracellular uptake and reducing the associated toxicity through control of the release rate. Hybrid systems incorporating Janus and dendrimer particles into composite materials show promise for improving the biocompatibility and drug delivery of pharmaceuticals, while overcoming the limitations of stand-alone nanoparticles. Janus–dendrimer particles require further investigation and fine-tuning to enter the clinical setting and improve therapeutic outcomes.

Urinary and central line catheters are prone to colonization by microorganisms in biological environments, which is one of the main causes of nosocomial infections in intensive care units. Duarte-Peña, Magaña and Bucio developed materials capable of simultaneously preventing bacterial adhesion and releasing an antibacterial drug, e.g., ciprofloxacin. These dual antimicrobial materials were produced by gamma-radiation-induced grafting of poly(vinyl chloride) catheters with 4-vinyl pyridine and later functionalization with 1,3-propane sultone. The surface properties of the materials were determined by thermogravimetric analysis, FTIR, swelling behavior, pH-responsiveness and contact angle measurements. The drug loading and release, inhibition of bacterial growth, bacterial and protein adhesion and cell growth stimulation were also evaluated. The hydrophilicity and pH sensitivity of the system were related to its ability to load and release ciprofloxacin. Reductions in bovine serum albumin and *E. coli* adhesion were enhanced by loading with ciprofloxacin. The modified materials were shown to be non-toxic, as the cell viability was not significantly affected. These hold promise for the manufacture of medical devices, such as catheters, for local prophylaxis and treatment of infections typically associated with their use, increasing their efficacy and reducing side effects.

Additive manufacturing offers the opportunity to design and develop innovative systems with complex geometries and programmed controlled release profiles that rely on the use of polymers [9]. In this next work, the antimicrobial efficacy of a novel 3D-printed scaffold containing vancomycin (Van) against Staphylococcus species was evaluated for use in drug delivery and tissue engineering. Local administration of the antibiotic has been shown to offer benefits over the traditional parenteral route in acute and chronic bone infections when ischemia is present. The medicated scaffold was based on polycaprolactone (PCL) and a chitosan (CS) hydrogel. The hydrophobicity of PCL (determined via contact angle measurements) was reduced by two cold plasma treatments to improve the adhesion of the CS hydrogel. The release of vancomycin was followed by high-performance liquid chromatography (HPLC). Surgical site and medical-device-associated infections are primarily caused by the opportunistic Gram-positive *Staphylococcus aureus* and *Staphylococcus epidermidis*—the microorganisms challenged in this study to evaluate the antibacterial efficacy. Finally, the biological response to the scaffolds was evaluated in terms of cytotoxicity (lactate dehydrogenase activity), proliferation and osteogenic differentiation (alkaline phosphatase activity; alizarin red staining) in a population of adult human bone-marrow-derived mesenchymal stem cells. The hybrid PCL/CS/Van scaffolds combined the biocompatibility, biodegradability and antibacterial properties of CS with the mechanical properties of PCL. Further in vivo preclinical studies using animal models are needed to confirm the usefulness of the systems in achieving a controlled and effective local release of vancomycin in bone infections.

Transformation of crystalline drugs into their more water-soluble amorphous counterparts may be achieved by producing amorphous solid dispersions (ASDs) with water-soluble polymeric carriers or co-amorphous dispersions (CADs) with low molecular weight entities, as presented in the next two papers. The advantages and disadvantages of each approach have recently been the subject of a review [10].

The molecular interactions established between delayed-release dugs and polymers used in the production of ASDs were investigated to simplify the development of such entities. At first, the compatibility of the drug and polymer in the solid state was investigated by molecular dynamics simulations, and used later on to identify ideal pairs. Afterwards, drug ASDs were produced by hot-melt extrusion (HME), an emergent processing technology for such purposes. In brief, a drug is dispersed in a polymeric matrix, heated at high temperatures (below the drug melting point) and mixed in an extruder to achieve homogeneity. The extrudates were milled and characterized by XRD, FTIR spectroscopy and dissolution studies. The potential of each drug–polymer pair considered was evaluated by determination of (a) the drug–polymer interaction energy (electrostatic, Lenard–Jones and total energies), (b) energy ratio (drug–polymer/drug–drug) and (c) drug–polymer hydrogen bonding. The best total energy values corresponded to naproxen–Eudragit^®^ L100 (−143.38 kJ/mol), diclofenac sodium–hydroxypropyl methylcellulose phthalate (−348.04 kJ/mol), dimethyl fumarate–hydroxypropyl methylcellulose acetate succinate (−110.42 kJ/mol) and omeprazole–hydroxypropyl methylcellulose acetate succinate (−269.43 kJ/mol). The energy ratio trends of the pairs were consistent with these results and every drug–polymer pair established hydrogen bonds. Few drug–polymer pairs were extrudable by HME (up to 50% drug load); the extrudates did not release the drug at pH 1.2 (simulated gastric fluid) but released it at pH 6.8 (simulated intestinal fluid). The study not only demonstrates drug–polymer compatibility, but also that each drug requires a different enteric polymer. The atomic-level insights gathered are essential for the rational design and screening of ASD formulations.

Mohamed and co-workers developed CADs of tacrolimus (an immunosuppressive drug with low solubility) and sucrose acetate isobutyrate (SAIB) as a co-former. The in vitro and in vivo performance of the system was compared with that of a hydroxypropyl methylcellulose (HPMC)-based ASD. The solvent evaporation method was used to prepare both CADs and ASDs, which were characterized by FTIR, XRD and differential scanning calorimetry (DSC) and evaluated for dissolution, stability and oral pharmacokinetics in beagle dogs. Amorphization of tacrolimus was demonstrated by XRD and DSC, and more than 85% of the drug was dissolved within 90 min. Drug dissolution could be modulated by changing the drug/excipient ratio. After short-term stability testing (storage at 25 °C/60% RH and 40 °C/75% RH), no drug crystallization was observed in the thermograms and diffractograms; the dissolution profiles before and after storage were similar, again indicating the stability of the amorphous systems. The two formulations, the SAIB-based CAD and the HPMC-based ASD, were considered bioequivalent regarding the Cmax and AUC, meeting the established FDA confidence interval of 90–111.1% for narrow therapeutic index drugs. Their Cmax and AUC values were respectively 1.7–1.8 and 1.5–1.8 times higher than that of crystalline drug tablet formulations; thus, they are expected to be more bioavailable. Long-term stability evaluations and human clinical trials are required to demonstrate their superior pharmacokinetics and clinical efficacy.

The future of drug delivery lies in the use of increasingly complex, biodegradable, biocompatible and intelligent polymers [11]. Further basic and applied research is required to achieve greater control over the properties and performance of polymer-based drug delivery and targeting systems to fulfill their great potential in pharmaceutics.

## Figures and Tables

**Figure 1:**
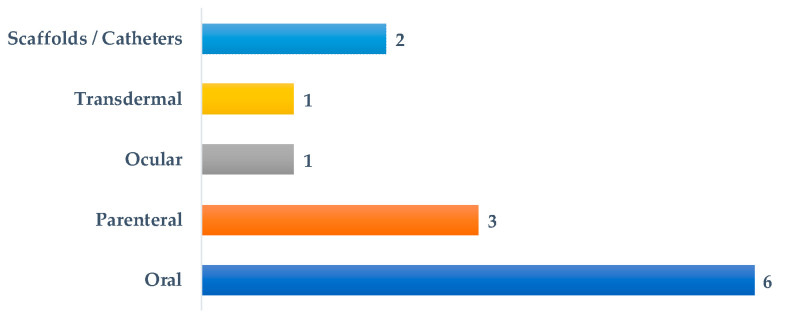
Number of papers published according to the route of administration or type of device.

## Data Availability

Data are contained within the article.
